# Изучение генетических факторов, определяющих признак «тетраостость» мягкой пшеницы

**DOI:** 10.18699/VJ20.650

**Published:** 2020-10

**Authors:** O.B. Dobrovolskaya, A.E. Dresvyannikova, E.D. Badaeva, K.I. Popova, M. Trávníčková, P. Martinek

**Affiliations:** Institute of Cytology and Genetics of Siberian Branch of the Russian Academy of Sciences, Novosibirsk, Russia RUDN University, Agrarian and Technological Institute, Moscow, Russia; Institute of Cytology and Genetics of Siberian Branch of the Russian Academy of Sciences, Novosibirsk, Russia; Vavilov Institute of General Genetics of the Russian Academy of Sciences, Moscow, Russia; Novosibirsk State Agricultural University, Novosibirsk, Russia; Crop Research Institute, Prague, Czech Republic; Agrotest Fyto, Ltd., Kroměříž, Czech Republic

**Keywords:** wheat, Triticum aestivum L., spike, awnedness, awned glume, molecular-genetic mapping, SEM, B1 awn suppressor, пшеница, Triticum aestivum L., колос, остистость, тетраостость, молекулярно-генетическое картирование, SEM, ингибитор остистости B1

## Abstract

Ости – тонкие заостренные отростки, сформированные в дистальной части чешуй колоска соцветия некоторых видов злаков, включая такие экономически значимые культуры, как пшеница мягкая (Triticum aestivum L.) и твердая (T. durum Desf.), ячмень (Hordeum vulgare L.), рис (Oryza sativa L.), рожь (Secale cereal L.). Наличие длинных остей на колосковых чешуях характерно для одного вида пшеницы – T. carthlicum Nevski, киль коло- сковой чешуи которого переходит в длинный остевидный отросток или ость, равную по длине ости цветковой чешуи. Колос T. carthlicum имеет удвоенное число остей, а сам признак получил название «тетраостость» или персикоидность. Ости на месте килевого зубца колосковых чешуй могут формироваться у пшениц T. aestivum и T. aethiopicum, однако такие формы встречаются редко. Особенности развития признака тетраостости и его гене- тические детерминанты изучены мало. В настоящем исследовании рассмотрены особенности развития и насле- дования признака «тетраостость» линии CD 1167-8 мягкой пшеницы T. aestivum c применением классического генетического анализа, молекулярно-генетического картирования и сканирующей электронной микроскопии. Показано, что признак наследуется как рецессивный моногенный. Ген, контролирующий тетраостость линии CD 1167-8, картирован в длинном плече хромосомы 5A c использованием 15K-SNP-микрочипа, содержащего 15 000 ассоциированных с генами SNP пшеницы (Illumina Infinium 15K Wheat Array, TraitGenetics GmbH). Резуль- таты теста на аллелизм продемонстрировали, что изучаемый ген аллелен b1, рецессивному аллелю гена-инги- битора остистости B1 (5AL). Таким образом, ген, контролирующий формирование остей на колосковых чешуях мягкой пшеницы, является рецессивным аллелем гена ингибитора остистости B1. Новый аллель обозначен b1.ag (b1. awned glume). Анализ развивающегося соцветия линии CD 1167-8 с помощью сканирующей электронной микроскопии выявил, что зачатки остей колосковых чешуй формируются по мере развития и роста колосковых чешуй одновременно с развитием остей на цветковых чешуях, различий в развитии остей на цветковых и колосковых чешуях не обнаружено.

## Abstract (en)

Awns are bristle‐like structures, typically extending from the tip end of the lemmas in the florets of cereal species, including such economically important crops as wheat (Triticum aestivum L., T. durum Desf.), barley (Hordeum vulgare L.), rice (Oryza sativa L.), and rye (Secale cereale L.). The presence of long awns adhered at tip end of glumes is a characteristic feature of “Persian wheat” T. carthlicum Nevski spike. Glume outgrowth of T. carthlicum Nevski spike passes into a long awn, equal in length to the lemma awn. Awned glumes can be formed in T. aestivum and T. aethiopicum wheats, however, such forms are rare. Features of the awned glume development and the genetic determinants of this trait have been little studied. In this paper, we described the features of the development and inheritance of the tetra-awness (awned glume) trait of the bread wheat T. aestivum line CD 1167-8, using classical genetic analysis, molecular genetic mapping, and scanning electron microscopy. It was shown that the trait is inherited as a recessive monogenic. The gene for the awned glume trait of CD 1167-8 was mapped in the long arm of chromosome 5A, using the Illumina Infinium 15K Wheat Array (TraitGenetics GmbH), containing 15,000 SNPs associated with wheat genes. Results of allelism test and molecular-genetic mapping suggest that the gene for awned glumes in bread wheat is a recessive allele of the B1 awn suppressor. This new allele was designated the b1.ag (b1. awned glume). Analysis of the CD 1167-8 inflorescence development, using scanning electron microscopy, showed that awns had grown from the top of the lemmas and glumes simultaneously, and no differences in patterns of their development were found.

## Введение

Ости представляют собой тонкие заостренные отростки, сформированные в дистальной части чешуй соцветий некоторых видов злаков, в том числе экономически значи- мых сельскохозяйственных культур – пшеницы (Triticum aestivum, T. durum), ячменя (Hordeum vulgare), риса (Oryza sativa) и ржи (Secale cereale).

У дикорастущих злаков основная функция остей – распространение плодов (зерновок). Кроме того, они выполняют защитную роль и препятствуют поеданию плодов животными и птицами. В процессе доместикации эти функции утратили свое значение. Доместикация сопро- вождалась редукцией органов, способствующих рас- пространению семян – остей, волосков, щетинок (Fuller, Allaby, 2018). У многих культивируемых сортов пшеницы и ячменя ости сохранились, однако, по сравнению с дико- растущими предками, стали короче, тоньше и легче (Peleg et al., 2010; Haas et al., 2019). Ости пшеницы, ячменя и ржи содержат хлоренхиму и являются фотосинтезирующими органами. Показано, что доля вклада остей в фотосин- тезе колоса ячменя составляет 70–90 %. При этом 30 % всех сухих веществ зерна, в том числе 50 % крахмала, создается остями (Ионова, 2005). Наличие остей может способствовать увеличению урожайности в определенных климатических условиях (засушливость, повышенные температуры). Остистые формы преобладают среди сортов пшениц Австралии, Южной и Центральной Амери- ки, США (Rebetzke et al., 2016). В условиях Северной и Центральной Европы остистость не обеспечивает адап- тивные преимущества и среди сортов пшеницы преобла- дают безостые формы (Börner et al., 2005; Rebetzke et al., 2016).

Наружная цветковая чешуя у мягкой пшеницы несет ость или острый зубец (изредка вершина тупая, без зуб- ца), на колосковых чешуях этого вида могут встречаться острые длинные (до 5 см) килевые зубцы (Дорофеев и др., 1979). У тетраплоидной пшеницы T. carthlicum Nevski (классификация В.Ф. Дорофеева (1979), синоним «Персидская пшеница» T. persicum Vav. – наименование вида, данное Н.И. Вавиловым) киль колосковой чешуи пере- ходит в длинный остевидный отросток или ость длиной до 12 см. Длина ости колосковой чешуи равна длине цветковой чешуи, и весь колос имеет удвоенное число остей. Наличие длинных остей на колосковых чешуях T. carthlicum – одна из основных характерных черт этого вида. Ости вместо килевого зубца колосковых чешуй могут формироваться у некоторых рас T. aestivum, однако у других видов пшениц встречаются крайне редко (Вави- лов, Якушкина, 1925). В.Ф. Дорофеев с коллегами (1979) отмечали наличие тетраостых колосьев у T. aethiopicum.

У мягкой пшеницы безостость доминирует над ости- стостью (Гончаров, 2012). Известны три доминантных неаллельных гена, ингибирующих развитие остей пше- ницы, B1, B2 и Hd, локализованные в хромосомах 5AL, 6BL и 4BS соответственно (Watkins, Ellerton, 1940; Sears, 1954, 1966; Kato et al., 1998; Sourdille et al., 2002; Yoshioka et al., 2017; Huang et al., 2020). Наиболее распространен аллель B1, он ингибирует развитие остей у гексаплоидных и тетраплоидных пшениц (Гончаров, 2002; Le Couviour et al., 2011; Mackay et al., 2014; Yoshioka et al., 2017). Для всех трех генов определена локализация в хромосомах с помощью молекулярно-генетического картирования (Yoshioka et al., 2017). Недавно D. Huang с коллегами (2020) показали, что ген B1 кодирует транскрипционный фактор с мотивом «цинковые пальцы» C2H2-типa. Анализ гаплотипов B1 показал, что доминантный аллель этого гена – наиболее распространенный ингибитор остей у пшеницы (Huang et al., 2020).

Признак «тетраостость» пшеницы изучен в меньшей степени. Н.И. Вавилов и О.В. Якушкина (1925) исследо- вали характер наследования ряда признаков, характерных для «Персидской пшеницы» T. carthlicum, среди которых был признак «длина остевидных придатков колосковых чешуй». Они провели масштабный гибридологический анализ, скрещивая T. carthlicum с различными видами ди-, тетра- и гексаплодных пшениц, а также эгилопсами и рожью (всего 64 комбинации скрещиваний), и изучили закономерности наследования признаков. Была отмечена сложная генетическая природа признака, показано, что он контролируется несколькими генами. В других работах был обнаружен рецессивный характер наследования при- знака тетраостости у T. carthlicum (Мигушова, Жуковский, 1969). По данным П.А. Гандиляна (1973), наличие тетраостости (персикоидности) связано со специфическим геном Т. Р.В. Рожков с коллегами (2014) показали, что признак «тетраостость» T. carthlicum и T. petropavlovskyi при скрещивании с сортами твердой (T. durum) и мягкой (T. aestivum) пшениц соответственно, наследуется как ре- цессивный. При этом, если при скрещивании T. petropavlovskyi с мягкой пшеницей за формирование тетраостости отвечает один ген, то в комбинациях с T. persicum влияние оказывают несколько генов.

Настоящее исследование посвящено изучению генети- ческого контроля и особенностей формирования признака «тетраостость» мягкой пшеницы. С использованием мо- лекулярно-генетического картирования была определена локализация главного гена, контролирующего тетраостый фенотип, в хромосоме 5AL мягкой пшеницы. Локализа- ция гена и результаты теста на аллелизм предполагают, что этот ген является рецессивным аллелем ингибитора остистости B1.

## Материалы и методы

**Растительный материал.** Исследовали линию мягкой пшеницы T. aestivum CD 1167-8 с тетраостым фенотипом, полученную д-ром П. Мартинеком (Агротест Фито Лтд., Кромержиж, Чешская Республика).

Изучение особенностей наследования признака «тетра- остость» проводили на гибридах F_1_, F_2_ и F_3_ от скрещива- ния CD 1167-8 и безостой линии мягкой пшеницы сорта Новосибирская 67 (Н67) и последующих самоопылений. Линия CD 1167-8 использована в скрещиваниях и как материнское, и как отцовское растение.

Тест на аллелизм проведен при скрещивании CD 1167-8 с остистой линией мягкой пшеницы Ruc 204, полученной д-ром П. Мартинеком. Эта линия была ранее детально охарактеризована с применением методов молекулярной генетики и цитогенетики (Добровольская, 2018). Все используемые в работе линии мягкой пшеницы имеют яровой тип развития.

Растения выращивали в полевых условиях на базе селекционно- генетического комплекса Института цитоло- гии и генетики СО РАН (г. Новосибирск, 2015–2016 гг.), а также в полевых условиях в г. Кромержиж (Чешская Республика, 2016 г.). Оценку фенотипов колоса (наличие/ отсутствие остей колосковых и цветковых чешуй) прово- дили после полного созревания растений. Соответствие фактического расщепления теоретически ожидаемому в популяциях гибридов оценивали по критерию χ2 (Рокиц- кий, 1973).

Молекулярно-генетическое картирование. Образцы суммарной ДНК выделяли из листьев 90 индивидуальных растений популяции F_2_ CD 1167-8 × Н67 (далее – картирующей популяции) и родительских линий CD 1167-8 и Н67, согласно методу J. Plaschke с коллегами (1995).

Генотипирование растений картирующей популяции выполняли с использованием 15K-SNP-микрочипа, содер- жащего 15 000 SNP, ассоциированных с генами пшеницы (Illumina Infinium 15K Wheat Array, TraitGenetics GmbH, Гатерслебен, Германия). Анализ полученных данных, по- строение молекулярно-генетической карты осуществля-ли при помощи программы MultiPoint версии UltraDense (Ronin et al., 2010, 2015), как описано ранее (Dresvyannikova et al., 2019). Графическое изображение молекулярно- генетической карты выполнено с помощью программы MapChart 2.2 (Voorrips, 2002).

**Сканирующая электронная микроскопия.** Развивающиеся соцветия линии CD 1167-8 вычленяли из вторичных побегов растений с использованием бинокулярного микроскопа Альтами ПС0745 («Aльтами», Санкт-Петербург, Россия). Особенности строения соцветия изучали при помощи сканирующего электронного микроскопа Hitachi TM-1000 (Hitachi, Ltd., Япония) при постоянном ускоряющем напряжении 15 кВ и степени разряжения в камере для образца 30–50 Па. Растительный материал для сканирующей электронной микроскопии не подвергали предварительной обработке. Для получения и обработки изображений использовали программное обеспечение для Hitachi TM-1000.

**Изучение кариотипа.** Кариотип тетраостой линии CD 1167-8 изучен с применением С-дифференциального окрашивания, проводенном по раннее опубликованной методике (Badaeva et al., 1994). Препараты анализи- ровали при помощи микроскопа Leitz Wetzlar (Leika microsystems, Германия). Для получения изображений использовали цифровую камеру CCD Leica DFC 280 (Leika microsystems). Хромосомы классифицировали в соответ- ствии со стандартной номенклатурой (Gill et al., 1991).

## Результаты и обсуждение

Линия мягкой пшеницы CD 1167-8 характеризуется на- личием остей как на цветковых, так и на колосковых чешуях (рис. 1, а, б ). Ости колосковых чешуй этой линии (2.5 ± 0.1 см) короче и тоньше остей цветковых чешуй. Признак проявлялся при выращивании растений линии в полевых условиях г. Новосибирска (2015–2016 гг.) и г. Кромержижа Чешской Республики (2016 г.), а также в условиях тепличного комплекса ИЦиГ СО РАН (2009– 2017 гг.). Признак стабильно наследовался при само- опылении линии. Таким образом, наличие тетраостости изучаемой линии стабильно наследуется и проявляется в различных условиях выращивания.

**Fig. 1.**
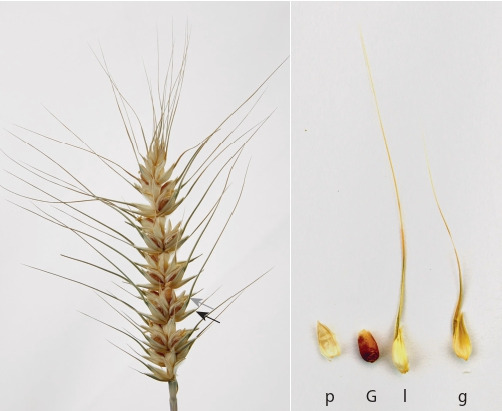
A four-awned glume spike of the bread wheat line CD 1167-8. Gray and black arrows designate awns of (l) lemma and (g) glume, respectively. G, grain; p, palea.

Изучение ранних этапов развития соцветия линии CD 1167-8 мягкой пшеницы с использованием сканирующей электронной микроскопии показало, что зачатки остей колосковых чешуй формируются по мере развития и роста колосковых чешуй одновременно с развитием остей на цветковых чешуях (рис. 2). Особенностей, отличающих развитие остей колосковых чешуй от развития остей цветковых чешуй, не обнаружено.

**Fig. 2.**
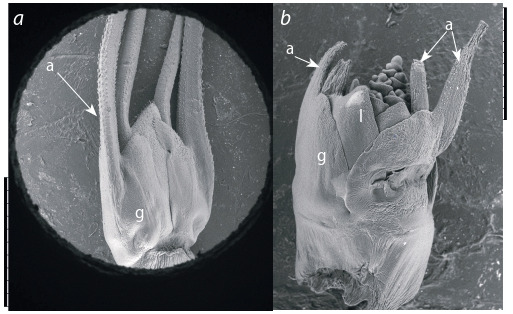
SEM analysis of a spikelet of a developing bread wheat inflorescence in line CD 1167-8. a – awns (a) on glumes (g) of a spikelet, scale bar 2 mm; b – the initial growth stage of glume and lemma (l) primordia, scale bar 1 mm.

Для выявления генетических детерминант тетраостости мягкой пшеницы были получены популяции гибридов от скрещиваний CD 1167-8 и безостой линии пшеницы Ново- сибирская 67 (Н67), в которых растение тетраостой линии CD 1167-8 использовано и как материнское (CD1167-8 × Н67), и как отцовское (Н67 × СD1167-8). Гибриды F_1_ были безостыми, а в поколении гибридов F_2_ наблюдали рас- щепление на безостые и остистые (тетраостые) формы с преобладанием безостых. Все остистые растения F_2_ были тетраостыми.

Из 117 растений гибридов F_2_ от скрещивания Н67 × СD 1167-8 34 были остистыми, а остальные 83 – безостыми, что соответствует моногенному рецессивному типу наследования признака «тетраостость» (χ^2^ = 1.028, p = 0.05). Аналогичные результаты получены и в скре- щивании CD 1167-8 × Н67 – соотношение остистых рас- тений (41) к безостым (119) соответствует 1 : 3 (χ^2^ = 0.033, p = 0.05), что означает моногенное рецессивное наследо- вание признака. Различий в реципрокных скрещиваниях не обнаружено.

При изучении наследования ряда характерных для «Персидской пшеницы» признаков Н.И. Вавилов и О.В. Якушкина (1925) отмечали, что развитие остевидных придатков на колосковых чешуях – хорошо наследуемый, мало зависящий от внешних условий признак. Обобщая результаты 62 комбинаций скрещиваний с различными видами пшениц, они сделали вывод о полимерном насле- довании признака. Вместе с тем отмечено, что при скре- щивании с безостыми формами безостость доминирует и в поколении гибридов F_2_ выщепляются остистые формы, составляющие 1/4 часть от всех гибридных растений. Остистые формы остаются константными в F_3_ (Вавилов, Якушкина, 1925). Рецессивный тип наследования под- твержден и другими учеными (Мигушова, Жуковский, 1969; Рожков и др., 2014). Кроме того, был обнаружен моногенный рецессивный тип наследования признака при скрещивании гексаплоидных видов пшениц (Рожков и др., 2014).

Результаты генетического анализа показали, что при- знак тетраостости у мягкой пшеницы также стабильно наследуется и находится под моногенным рецессивным контролем. Обращающей на себя внимание особенностью стало совместное наследование остистости колосковых и цветковых чешуй.

Локализация гена, контролирующего признак «тетраостость » у мягкой пшеницы, была определена с применением молекулярно-генетического картирования на субпопуляции F_2_ CD 1167-8 × Н67, включающей 90 рас- тений. Из 90 растений картирующей популяции F_2_ 28 были остистыми (тетраостыми), остальные – безостыми. При самоопылении гибридов F_2_ получены 90 семей ги- бридов F_3_, анализ которых проводили в полевых усло- виях г. Новосибирска (1127 растений) и г. Кромержижа (954 растения). Анализ гибридов F_3_ показал, что 28 семей от самоопыления тетраостых гибридов F_2_ проявляли при- знак «тетраостость», а в семьях от безостых гибридов на-блюдалось либо расщепление на остистые и безостые фор- мы (43 семьи), либо потомки были безостыми (19 семей). Расщепление семей F_3_ подтверждает моногенный рецес- сивный характер наследования признака 1 : 2 : 1 (χ^2^ = 1.97, p < 0.05). Признак «тетраостость» несколько варьировал в своем проявлении, но ни одного растения с парой остей, расположенных исключительно на цветковых или только на колосковых чешуях, не обнаружено.

Для молекулярно-генетического картирования использовали данные высокопроизводительного генотипиро- вания. Всего проанализировано 5160 информативных SNP-локусов. Показано сцепление генетического локуса, контролирующего формирование тетраостого фенотипа, с маркерами хромосомы 5A (рис. 3, см. Приложение)^1^. Изучаемый ген расположен дистально по отношению к SNP-маркеру BS00023138-51 (на расстоянии 3 сМ).

**Fig. 3.**
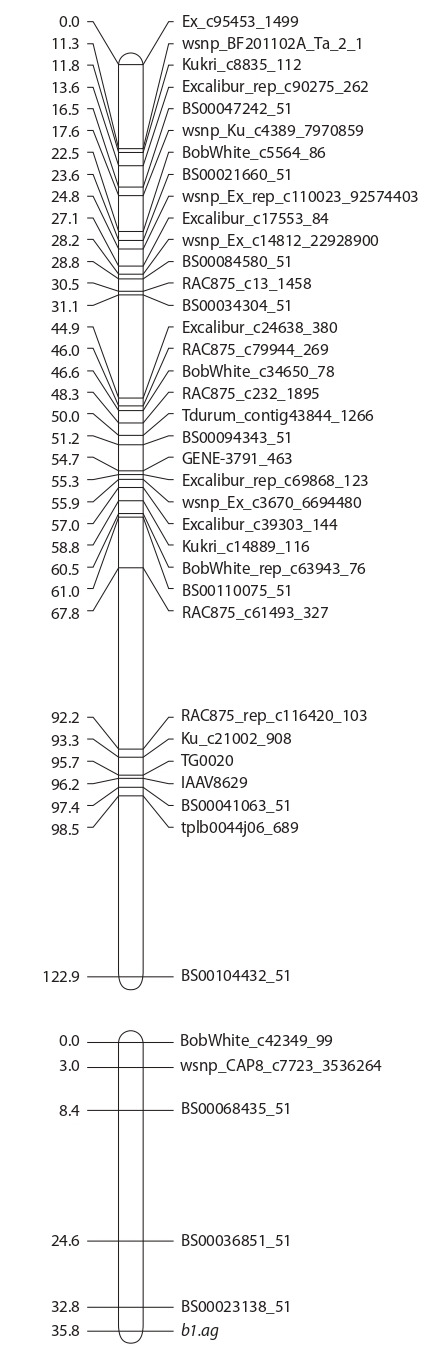
A molecular map of chromosome 5A involving the b1.ag gene for the awned glume of bread wheat. Distances in cM are shown on the left, and SNP markers are shown on the right (only skeleton markers representing groups of cosegregating markers are shown).

^1^Приложение см. по адресу: http://www.bionet.nsc.ru/vogis/download/pict-2020-24/appx8.pdf

Известно, что хромосома пшеницы 5AL несет доми- нантный ген-ингибитор развития остей – B1. Локализация гена, определяющего тетраостость, и гена B1 в дисталь- ном районе хромосомы 5AL совпадает (Yoshioka et al., 2017). На основании этого можно предположить, что тетраостость определяется рецессивным аллелем гена B1, который отличается от рецессивного аллеля, распростра- ненного у сортов мягкой и твердой пшениц и приводящего к развитию пары остей на цветковых чешуях.

Далее был выполнен тест на аллелизм, линия CD 1167-8 с тетраостым колосом была скрещена с линией Ruc 204 c типичным для T. aestivum остистым колосом (ости раз- виваются только на цветковых чешуях). Анализ фенотипа гибридов F_1_ показал, что все они были остистыми и имели ости как на цветковых, так и на колосковых чешуях. Таким образом, наличие ости на колосковой чешуе до-минировало над отсутствием, однако длина ости у гибридов (1.4 ± 0.1 см) была короче, чем у тетраостого родителя (2.5 ± 0.1). Среди гибридов поколения F_2_ совершенно без- остых растений не обнаружено, все растения (176) имели ости на цветковых чешуях; на колосковой чешуе ости длиной 1–4 см обнаружены у 93 растений, у 83 растений ости на колосковой чешуе не достигали длины 0.5 см, у 4 растений ости на колосковых чешуях полностью от- сутствовали, как у линии Ruc 204.

Полученные результаты указывают на то, что ген, контролирующий тетраостый фенотип мягкой пшеницы, аллелен гену-ингибитору остистости B1 и является его ре- цессивным аллелем. Этот аллель мягкой пшеницы описан нами впервые и обозначен как b1.ag (b1. awned glume).

Выявлено, что отсутствие остей под контролем гена- ингибитора остистости B1 доминирует над их наличием (b1 и b1.ag), при этом наличие остей на колосковой чешуе под контролем аллеля b1.ag доминирует над отсутствием под контролем аллеля b1. При этом характер расщепления признака «остистость колосковых чешуй», обнаружен- ный при скрещивании тетраостой CD 1167-8 и остистой Ruc 204 линий пшеницы, более соответствует количественному наследованию признака, следовательно, в контроле изучаемого признака наряду с b1.ag принимают участие и другие гены.

Н.И. Вавилов и О.В. Якушкина (1925) показали, что на особенности наследования остистости колосовых чешуй в комбинациях скрещиваний «Персидской пшеницы» с другими видами пшениц могут оказывать влияние не только наличие или отсутствие коротких (до 2 мм) остей у второго родителя, но и форма, ширина и другие особен- ности колосковых чешуй, что предполагает участие и других генов в контроле изучаемого признака.

Тетраостость не распространена широко среди об- разцов мягких пшениц. Н.И. Вавилов и О.В. Якушкина (1925) отмечали, что тетраостый фенотип, сходный с фенотипом «Персидской пшеницы», встречается среди туркестанских, персидских и бухарских мягких пшениц. Вместе с тем характерным (систематическим) признаком «тетраостость» является только для одного вида пшениц – T. carthlicum.

Кариотип линии CD 1167-8 изучен с использованием дифференциального С-окрашивания хромосом с целью выявления возможных сегментов интрогрессии от дру- гих видов пшеницы. Показано, что линия CD 1167 имеет кариотип, характерный для мягкой пшеницы (см. рис. 4). Вероятно, b1.ag – это редкий аллель гена B1, встречаю- щийся у мягкой пшеницы.

**Fig. 4.**
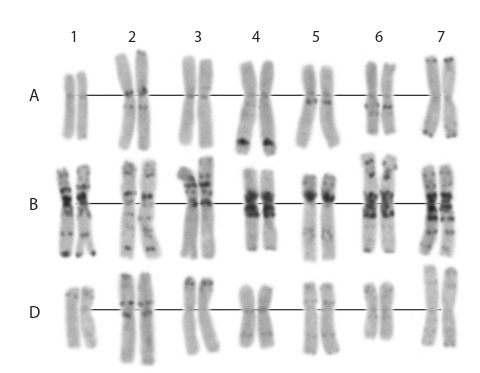
Karyotype of the CD 1167-8 line (C-banding).

## Заключение

Тетраостость, или развитие остей на колосковых че- шуях у мягкой пшеницы, наследуется как рецессивный моногенный признак и находится под контролем гена, локализованного в хромосоме 5AL. Обобщая результаты проведенного нами генетического анализа и молекулярно- генетического картирования, можно предположить, что ген тетраостости мягкой пшеницы является рецессивным аллелем ранее изученного гена-ингибитора остистости B1. Этот аллель описан нами впервые и обозначен как b1.ag (b1.awned glume). Показано, что отсутствие остей под контролем гена-ингибитора остистости B1 доминирует над их наличием (b1 и b1.ag), при этом наличие остей на колосковой чешуе под контролем аллеля гена b1.ag доминирует над отсутствием под контролем аллеля b1. Различий в развитии остей на колосковых и цветковых чешуях не обнаружено.

## Conflict of interest

The authors declare no conflict of interest.

## References

Вавилов Н.И., Якушкина О.В. К филогенезу пшениц. Гибридоло-
гический анализ вида T. persicum Vav. и межвидовая гибридизация у пшениц. Тр. по прикл. бот., ген. и селекции. 1925;15(1):
3-159.
[Vavilov N.I., Yakushina O.V. On the phylogenesis of wheat. Test
cross analysis of T. persicum Vav. and interspecific hybridization of
wheat. Trudy po Prikladnoy Botanike, Genetike i Selektsii
= Proceedings on Applied Botany, Genetics, and Breeding. 1925;15(1);
3-159. (in Russian)]

Гандилян П.А. Колосовые культуры и их дикие сородичи Армянской ССР: Автореф. дис. … д-ра биол. наук. Ереван, 1973.
[Gandilyan P.A. Spike crops and their wild relatives of the Armenian
SSR: Doctor Sci. (Biol.) Dissertation. Yerevan, 1973. (in Russian)]

Гончаров Н.П. Сравнительная генетика пшениц и их сородичей.
Новосибирск: Сиб. унив. изд-во, 2002.
[Goncharov N.P. Comparative Genetics of Wheats and their Related
Species. Novosibirsk: Siberian University Press, 2002. (in Russian)]

Гончаров Н.П. Сравнительная генетика пшениц и их сородичей.
Изд. 2-е испр. и доп. Новосибирск: Акад. изд-во «Гео», 2012.
[Goncharov N.P. Comparative Genetics of Wheats and their Related
Species. Novosibirsk: Geo Publ., 2012. (in Russian)]

Добровольская О.Б. Молекулярно-генетические основы морфогенеза соцветия пшеницы: Автореф. дис. … д-ра биол. наук. Новосибирск, 2018.
[Dobrovolskaya O.B. Molecular basis of wheat inflorescence morphogenesis:
Doctor Sci. (Biol.) Dissertation. Novosibirsk, 2018. (in Russian)]

Дорофеев В.Ф., Филатенко А.А., Мигушова Э.Ф., Удачин Р.А.,
Якубцинер М.М. Культурная флора СССР. Т. 1. Пшеница. Л.:
Колос, 1979.
[Dorofeev V.F., Filatenko A.A., Migushova E.F., Udachin R.A.,
Yakubtsiner M.M. The Cultural Flora of the USSR. Vol. 1. Wheat.
Leningrad: Kolos Publ., 1979. (in Russian)]

Ионова Н.Э. Роль отдельных органов в продукционном процессе
растений яровой пшеницы разного эколого-географического происхождения:
Автореф. дис. … канд. биол. наук. Спб., 2005.
[Ionova N.E. The role of individual organs in the production of
spring wheat plants of different ecological and geographical origins:
Cand. Sci. (Biol.) Dissertation. St. Petersburg, 2005. (in Russian)]

Мигушова Э.Ф., Жуковский П.М. К познанию пшеницы T. ispahanicum
Heslot. Тр. по прикл. бот., ген. и селекции. 1969;39(3):
71-90.
[Migushova E.F., Zhukovsky P.M. Towards the knowledge of wheat
T. ispahanicum Heslot. Trudy po Prikladnoy Botanike, Genetike i
Selektsii = Proceedings on Applied Botany, Genetics, and Breeding.
1969;39(3):71-90. (in Russian)]

Рожков Р.В., Криворученко Р.В., Коваленко И.В. Генетический
контроль тетраостости у пшеницы. Вестн. Харьковского национального аграрного университета. 2014;2(32):70-76.
[Rozhkov R.V., Krivoruchenko R.V., Kovalenko I.V. Genetic control
of tetrabeardedness in wheat. Vestnik Khar’kovskogo Natsional’nogo
Agrarnogo Universiteta = Bulletin of the Kharkiv National Agrarian
University. 2014;2(32):70-76. (in Ukrainian)]

Рокицкий П.Ф. Биологическая статистика. Минск: Вышейш. шк.,
1973;77-79.
[Rokitskii P.F. Biological Statistics. Minsk: Vysheishaya Shkola
Publ., 1973;77-79. (in Russian)]

Badaeva E.D., Badaev N.S., Gill B.S., Filatenko A.A. Intraspecific
karyotype divergence in Triticum araraticum (Poaceae). Plant Syst.
Evol. 1994;192(1-2):117-145. DOI 10.1007/BF00985912.

Börner A., Schäfer M., Schmidt A., Grau M., Vorwald J. Associations
between geographical origin and morphological characters in bread
wheat (Triticum aestivum L.). Plant Genet. Resour. 2005;3(3):360-
372. DOI 10.1079/PGR200589.

Dresvyannikova A.E., Watanabe N., Muterko A.F., Krasnikov A.A.,
Goncharov N.P., Dobrovolskaya O.B. Characterization of a dominant
mutation for the liguleless trait: Aegilops tauschii liguleless
(Lg t ). BMC Plant Biol. 2019;19(1):55. DOI 10.1186/s12870-019-
1635-z.

Fuller D.Q., Allaby R. Seed dispersal and crop domestication: shattering,
germination and seasonality in evolution under cultivation.
Annu. Plant Rev. Online. 2018;238-295. DOI 10.1002/9781119312
994.apr0414.

Gill B.S., Friebe B., Endo T.R. Standard karyotype and nomenclature
system for description of chromosome bands and structural aberrations
in wheat (Triticum aestivum). Genome. 1991;34(5):830-839.
DOI 10.1139/g91-128.

Haas M., Schreiber M., Mascher M. Domestication and crop evolution
of wheat and barley: Genes, genomics, and future directions.
J. Integr. Plant Biol. 2019;61(3):204-225. DOI 10.1111/jipb.
12737.

Huang D., Zheng Q., Melchkart T., Bekkaoui Y., Konkin D.J.F., Kagale
S., Martucci M., You F.M., Clarke M., Adamski N.M., Chinoy
C., Steed A., McCartney C.A., Cutler A.J., Nicholson P., Feurtado
J.A. Dominant inhibition of awn development by a putative
zinc‐finger transcriptional repressor expressed at the B1 locus in
wheat. New Phytol. 2020;225:340-355. DOI 10.1111/nph.16154.

Kato K., Miura H., Akiyama M., Kuroshima M., Sawada S. RFLP
mapping of the three major genes, Vrn1, Q and B1, on the long arm
of chromosome 5A of wheat. Euphytica. 1998;101(1):91-95. DOI
10.1023/A:1018372231063.

Le Couviour F., Faure S., Poupard B., Flodrops Y., Dubreuil P., Praud S.
Analysis of genetic structure in a panel of elite wheat varieties
and relevance for association mapping. Theor. Appl. Genet. 2011;
123(5):715-727. DOI 10.1007/s00122-011-1621-9.

Mackay I.J., Bansept-Basler P., Barber T., Bentley A.R., Cockram J.,
Gosman N., Greenland A.J., Horsnell R., Howells R., O’Sullivan
D.M., Rose G.A., Howell P.J. An eight-parent multiparent advanced
generation inter-cross population for winter-sown wheat:
creation, properties, and validation. G3 (Bethesda). 2014;4(9):1603-
1610. DOI 10.1534/g3.114.012963.

Peleg Z., Saranga Y., Fahima T., Aharoni A., Elbaum R. Genetic control
over silica deposition in wheat awns. Physiol. Plant. 2010;140(1):
10-20. DOI 10.1111/j.1399-3054.2010.01376.x.

Plaschke J., Ganal M.W., Röder M.S. Detection of genetic diversity
in closely related bread wheat using microsatellite markers. Theor.
Appl. Genet. 1995;91(6-7):1001-1007. DOI 10.1007/BF00223912.

Rebetzke G.J., Jimenez-Berni J.A., Bovill W.D., Deery D.M.,
James R.A. High-throughput phenotyping technologies allow accurate
selection of stay-green. J. Exp. Bot. 2016;67(17):4919-4924.
DOI 10.1093/jxb/erw301.

Ronin Y., Mester D., Minkov D., Korol A. Building reliable genetic
maps: different mapping strategies may result in different maps. Nat.
Sci. 2010;2(6):576-589. DOI 10.4236/ns.2010.26073.

Ronin Y., Minkov D., Mester D., Akhunov E., Korol A. Building ultradense
genetic maps in the presence of genotyping errors and missing
data. In: Advances in Wheat Genetics: from Genome to Field: Proc.
of the 12th Int. Wheat Genetics Symposium. Springer Nature, 2015;
127-133. DOI 10.1007/978-4-431-55675-6.

Sears E.R. The aneuploids of common wheat. Missouri Agr. Expt. Stn.
Res. Bull. 1954;572:1-59.

Sears E.R. Nullisomic-tetrasomic combinations in hexaploid wheat. In:
Riley R., Lewis K.R. (Eds.). Chromosome Manipulations and Plant
Genetics. Springer, Boston, MA. 1966;29-45. DOI 10.1007/978-1-
4899-6561-5_4.

Sourdille P., Cadalen T., Gay G., Gill B., Bernard M. Molecular and
physical mapping of genes affecting awning in wheat. Plant Breed.
2002;121(4):320-324. DOI 10.1046/j.1439-0523.2002.728336.x.

Voorrips R.E. MapChart: software for the graphical presentation of
linkage maps and QTLs. J. Hered. 2002;93(1):77-78. DOI 10.1093/
jhered/93.1.77.

Watkins A.E., Ellerton S. Variation and genetics of the awn in Triticum.
J. Genet. 1940;40(1-2):243-270.

Yoshioka M., Iehisa J.C.M., Ohno R., Kimura T., Enoki H., Nishimura
S., Nasuda S., Takumi S. Three dominant awnless genes in common
wheat: Fine mapping, interaction and contribution to diversity
in awn shape and length. PLoS One. 2017;12(4):e0176148. DOI
10.1371/journal.pone.0176148.

